# Sexual dimorphism, phenotypic integration, and the evolution of head structure in casque‐headed lizards

**DOI:** 10.1002/ece3.3356

**Published:** 2017-09-25

**Authors:** Gregory W. Taylor, Juan C. Santos, Benjamin J. Perrault, Mariana Morando, Carlos Roberto Vásquez Almazán, Jack W. Sites

**Affiliations:** ^1^ Department of Biology Bean Life Science Museum Brigham Young University Provo UT USA; ^2^ Department of Biological Sciences St. John's University Queens NY USA; ^3^ IPEEC‐CENPAT‐CONICET, U9120ACD Puerto Madryn Argentina; ^4^ Museo de Historia Natural Escuela de Biología Universidad de San Carlos de Guatemala Ciudad de Guatemala Guatemala

**Keywords:** biomechanics, Corytophanidae, geomorphometrics, head ornamentation, sexual dimorphism

## Abstract

Sexes can differ in features associated with differential reproduction, which can be used during courtship or aggressive encounters. Some traits tend to evolve independently between sexes and emerge as sexually dimorphic within the organismal phenotype. We characterize such a relationship by estimating the phenotypic integration of the head morphology and modularity of the crest in the casque‐headed lizards (Corytophanidae). In this clade, some species show extreme sexual dimorphism (e.g., head crests in the genus *Basiliscus*) while in others, both sexes are monomorphic. To characterize these patterns, we define phenotypic integration at the interspecific level as a pattern or network of traits evidenced by phylogenetically adjusted correlations that persist among species. At this level, modularity is an increased connectedness (e.g., higher correlation) among sections of these networks that persist in a lineage during the evolution of complex phenotypes. To test both concepts, we used phylogenetic geomorphometrics to characterize the head structure of corytophanid lizards, based on a time‐calibrated phylogeny that includes candidate fossil ancestors. We found evidence of an older diversification of corytophanids than previously reported (~67 vs. ~23.5 MYA) and show that this clade includes two morphological head architectures: (1) Sexually dimorphic crests present in males that are evolving independently from the rest of the head structure, and (2) full integration of the head morphology in monomorphic species. We propose that both architectures are optimal evolutionary trajectories of the parietal crest bones in the head of these lizards. In sexually dimorphic species, these bones are elongated and thinner, and gave rise to the extended crest used in male courtship displays. In monomorphic species, the parietal crest grew thicker in both sexes to allow for a better insertion of muscles associated with a stronger bite.

## 1 INTRODUCTION

Sexual dimorphism is common and is usually evidenced by traits that are more developed or exaggerated (e.g., larger or ornamented) in one particular sex (Shine, 1989). The evolutionary basis of such dimorphic phenotypes has usually been associated with fitness and resource availability. In males, for example, larger body size often correlates with territory area, while ornamentation might correlate with status and quality (Olsson, Shine, Wapstra, Ujvari, & Madsen, 2002). For females, larger mass is associated with condition and fecundity as proxies for the capacity to produce and hold developing embryos (Cox, Skelly, & John‐Alder, 2003). However, most of these studies are centered on the presence of sexual dimorphism and its fitness consequences, but rarely on the modularity of dimorphic traits with the rest of the organismal phenotype. Sexual dimorphism is usually assumed to originate from monomorphic ancestors, and sexual selection (e.g., female choice) drives the evolution of a particular set of traits through differential reproduction (Jones & Ratterman, 2009). Yet, the emergence of sexual dimorphism is an evolutionary trajectory evidenced by a set of traits that become exaggerated and, hence, modular to the rest of the phenotype in otherwise monomorphic species. Testing such evolutionary phenomena requires phylogenetic analyzes above the species level, where sexual dimorphism is expressed as distinctive phenotypic modules in one sex, and exploration of how it integrates (or not) with the organismal phenotype in the context of monomorphic relatives. However, we need operative definitions of phenotypic integration and modularity, which are useful for characterizing sexual dimorphism at the interspecific level.

Both concepts (i.e., phenotypic integration and modularity) have a rather difficult interpretation and usually combine intra‐ and interspecific perspectives (Abbott & Svensson, 2008; Pigliucci, 2003). At the intraspecific level, phenotypic integration usually refers to patterns of interdependence between genetic, developmental, and functional features among individuals (Olson & Miller, 1958). In this context, phenotypic integration uses multivariate studies of the phenotypic and genetic correlation/covariance assessed across members in a population (i.e., an observable pattern) and related discussions of linkage and pleiotropy as underlying mechanisms (Hallgrimsson et al., 2009). At this level, modularity as a concept is the division or grouping of traits observed among individuals in a population, usually through characterization such as developmental and genetic parcellation (i.e., differential gene expression). This pattern is evidenced as higher clustering or shared connections between traits than those outside this phenotypic module (Klingenberg, 2008, 2009). Historically, these definitions of integration and modularity are more common in the literature without a phylogenetic context, especially when referred to the way developmental processes were shaped by evolution, that is an evo‐devo perspective (Irschick et al., 2013).

At the interspecific level, phenotypic integration and modularity are more recent concepts, which we used in this study to characterize sexual dimorphism. For instance, both definitions are framed in a phylogenetic characterization of patterns of correlated evolution among traits that have high intraspecific interdependence (e.g., floral structure (Ordano, Fornoni, Boege, & Dominguez, 2008), postcranial skeletal morphology in mammals (Goswami, Smaers, Soligo, & Polly, 2014), aposematism in poison frogs (Santos & Cannatella, 2011), among others). Consequently, interspecific phenotypic integration, as an operational concept, is a pattern or network of traits evidenced by phylogenetically adjusted correlations that persist among species or clades over a long evolutionary time. The integrated phenotype is, consequently, inherited from ancestors to descendants and, during this process, it is modified (e.g., new components are integrated) or disrupted (e.g., loss of functionally or covariation between traits). Convergence and parallelism can explain how similarly functioning phenotypes originate on distantly related clades by tracing the correlations of its individual components as evidence of phenotypic integration. Likewise, interspecific modularity is the expected outcome of selection during evolution of complex or specialized phenotypes. For instance, a set of traits that share developmental and functional dependence will increase their connectedness (i.e., integration) as a result of directional selection along the history of a clade whose extant species present the same complex phenotype. In the case of sexual dimorphism, only one sex might present a set of highly correlated traits that can be associated with phenotypic integration by their prevalence in close relatives.

In practice, comparative geomorphological analyzes can be used to address these patterns of interspecific phenotypic integration and modularity by accounting for phylogenetic signal. These approaches can also add a temporal (phylogenetic comparative) perspective on the evolution of sexual dimorphism by comparison among close relatives. Even though sexual differences have been documented in many vertebrate clades, including feather coloration in Birds‐of‐Paradise (Paradisaeidae), antlers in cervids, and dewlaps among *Anolis* lizards (Davis, Brakora, & Lee, 2011; McGraw & Hill, 2000; Perry, 1996), to our knowledge, studies on both sexual dimorphism and phenotypic integration are rare (Pigliucci & Preston, 2004).

Reptiles are no exception to sexual dimorphism. Some examples include the larger body sizes in species in which reproduction is skewed to a few dominant males (e.g., lizards), or to females with larger capacity to harbor eggs (e.g., turtles) (Cox, Butler, & John‐Alder, 2007). Among Squamate (scaled) reptiles, specifically in the clade that includes lizards, two of the well‐known phenotypes associated with gender dimorphism are body size (e.g., snout‐vent length, SVL) and ornamentation (Olsson & Madsen, 1998). Larger body sizes are usually associated with aggressive and territorial species for which resources are limited, and the opportunities of reproduction are skewed toward fewer individuals (Blanckenhorn, 2005; Cox et al., 2003). In contrast, ornamentation is used as a signal of status, and it can be expressed in diverse forms, including showy color patterns in dimorphic structures (e.g., dewlaps). Among these forms are exaggerated crests or fins that increase perceived size and status of an individual by its competitors and potential mates (Olsson et al., 2002).

Here, we explored the evolutionary trajectory of one of these presumed ornamental structures: The head crest in casque‐headed lizards (Bohm et al., 2013; Cooper & Vitt, 1989), by considering them as a set of traits that are integrated and evolving as a module within a general pattern of morphological evolution across species. The “casque‐headed” lizards comprise the family Corytophanidae, a small clade of three genera (*Basiliscus*,* Corytophanes*, and *Laemanctus*) with nine extant species (Conrad, 2015; Vieira, Colli, & Bao, 2005). Among corytophanids, most males in *Basiliscus* present extreme head and body dimorphisms (e.g., large head crests and sail fins) that have been hypothesized as signals of status and aggressiveness. In contrast, males and females of *Corytophanes* and *Laemanctus* do not present evident anatomical differences, and sexual dimorphism appears to be restricted only to body size. However, these observations are based on qualitative taxonomic descriptions (Lang, 1989) and have never been tested by accounting for phylogenetic signal. Using geometric morphometrics, we characterized the head morphology of corytophanids in terms of individual traits (i.e., landmarks) that describe spatial features of the head, which can then be tested for interspecific phenotypic integration and modularity as they are related to sexual dimorphism.

For this purpose, we estimated a time‐calibrated phylogeny including fossil ancestors and traced the sexual differences in head morphology among all extant corytophanid species. Our objectives are to (1) re‐estimate a family level phylogeny using both molecular and morphological characters from extant and fossil species (only morphological characters for the latter), (2) compare the differences between size and shape of the cranial features between sexes at the interspecific level, and (3) quantify geomorphometric differences of the cranial features as evidence of interspecific phenotypic integration and modularity, to explain sexual dimorphism in casque‐headed lizards.

## 2 MATERIALS AND METHODS

### 2.1 Specimen measurements for geomorphometric analyzes

We measured a total of 286 specimens representing the nine extant species of Corytophanidae (Table S1). Specimen sex was determined by the presence/absence of hemipenes and, when possible, we included a balanced number of both sexes; this was not possible for some species (e.g., both *Laemanctus*) due to their rarity in US‐based collections (Table 1). We also excluded 19 specimens for which sex either could not be identified with certainty (body size <15 cm in length, considered juveniles), or were poorly fixed vouchers (e.g., the mouth widely agape or missing body parts). The remaining 261 specimens were assigned individual codes (Table S1), and snout‐vent lengths (SVL) were taken for all of these. The head of each specimen was photographed from three different viewpoints: dorsal, ventral, and a right‐side view of the cranium. Dewlap and cranial crests were extended and photographed for each individual that expressed those features (only the genus *Basiliscus*). All specimen images were submitted to Morphobank (http://www.morphobank.org), and the accession number of this project is as follows: P2602. An eight‐centimeter‐long forensic ruler (ABFO N#2, Crimetech.net, USA) was placed ventral to the head in each photo as a reference for size measurements, and we assigned 12 landmarks on the right‐side view of the head that included the crest in relationship to the face of the specimen (Fig. S1). Each image and its landmark information were digitized into a two‐dimensional coordinate TPS format file using TPSUtil (Rohlf, 2007) and TPSDig ver 2.05 (Rohlf, 2005). Relative measurements between landmarks were estimated using IMP ver 7 CoordGen (Sheets, 2010). After measurements were completed, all data were saved as text files for further geometric morphometric analyzes (Supplementary Script Appendix).

**Table 1:** Intraspecific differences on head morphology among casque‐headed lizards. Bold numbers indicate a p‐value < 0.05

Species	*N* ♂	*N* ♀	SVL pair *t*‐test *p*‐value	Procrustes ANOVA: shape by sex (no allometry)		Procrustes ANOVA: shape by sex (with allometry)				Sex disparity ratio (allometry correction) ♂ vs. ♀
				Sex		log(size)		Sex		
				*F*	*p*‐Value	*F*	*p*‐Value	*F*	*p*‐Value	
*Basiliscus plumifrons*	13	12	**.004**	18.110	**.001**	15.121	**.001**	5.631	**.001**	1.680
*Basiliscus basiliscus*	10	10	**.001**	15.673	**.001**	15.439	**.001**	0.934	.391	2.082
*Basiliscus galeritus*	9	9	.195	3.239	**.027**	6.608	**.001**	1.107	.275	0.407
*Basiliscus vittatus*	20	20	**.001**	45.849	**.001**	39.651	**.001**	6.912	**.001**	1.501
*Corytophanes cristatus*	16	20	.229	1.198	.279	2.669	**.026**	0.636	.634	1.246
*Corytophanes hernandesii*	5	25	.066	1.091	.301	3.481	**.008**	1.054	.309	0.682
*Corytophanes percarinatus*	10	41	.262	0.659	.717	3.024	**.013**	0.652	.714	0.769
*Laemanctus longipes*	2	13	—	1.057	.347	1.334	.222	1.300	.186	—
*Laemanctus serratus*	3	48	—	0.945	.449	0.893	.498	0.990	.397	—

### 2.2 Time‐calibrated phylogeny estimation

The phylogeny was reconstructed using both molecular and morphological characters from a total of 46 taxa including fossils (Table S2). The molecular data for corytophanid lizards included the NADH1‐tRNAs:IQM‐NADH2‐tRNAs:WANCY section (1781 bp) of the mitochondrial genome from 39 individuals (Table S3). Of these, 18 samples were new and derived from exome‐capture procedure with mitochondrial gene sequence baits, but only the NADH1‐tRNAs:IQM‐NADH2‐tRNAs:WANCY section was used in these analyzes (NCBI numbers: MF624292‐MF624309). Sequences of the other 21 samples were obtained from GenBank, and all accession numbers are given in Table S2. We used a total of 803 morphological characters from osteological, muscular, and gross anatomical descriptions (for character definitions see Tables S4 and S5). Seven species did not have molecular data, which include one extant species, *Laemanctus serratus*, and six fossil species that were used to calibrate the tree (for estimate ages see Table S4). Only osteological data were available from these fossil species.

Sequence alignment of each gene was performed using SATe ver 2.2.7 (Liu, Raghavan, Nelesen, Linder, & Warnow, 2009), and sections with large missing data were excluded. Models of molecular evolution for the tRNAs and codon positions of each gene were determined using jModelTest v 0.1.1 (Posada, 2008), and the selected molecular models are provided in Table S3. The final concatenated molecular and morphological matrix included a total of 1,690 molecular and 803 morphological characters (see Supplementary Data Appendix).

This matrix was used to estimate a maximum‐likelihood (ML) phylogeny using Garli ver 2.0 (Zwickl, 2006) and 200 nonparametric bootstrap searches estimated the nodal support. Our best topology did not differ from previous Bayesian and Parsimony analyzes of Corytophanidae that included only extant Pleurodont outgroups (Vieira et al., 2005). A chronogram of this clade was estimated using the best ML topology under a penalized likelihood rate smoothing (PLRS) approach with r8s ver 1.7 (Sanderson, 2002). Nodal age for the calibration of the PLRS guide chronogram was derived from our six corytophanid and closely allied fossils (Table S4). The final tree was estimated with the following options: after 20 random starts; with “checkgradient” option activated; penalty function as additive; optimization parameters under TN routine; smoothing parameter set at 10^*t*^ where *t *=* *0 from *t, t *+* *1, …, *t *+* *9 for cross‐validation; and local perturbation and fractional tolerance set to 0.01. The best‐score PLRS chronogram (Fig. S2 and Supplementary tree file) was used for all subsequent analyzes.

### 2.3 Statistical analyzes and modeling

All geometric morphometric analyzes were implemented in the R‐package “geomorph” ver 3.0.2 (Adams, Collyer, Kaliontzopoulou, & Sherratt, 2016) and custom R‐scripts derived from geomorph functions (see Supplementary Script Appendix). We read the TPS files with the list of its classifiers (e.g., genus, species, sex, and maturity) as a 2D‐array using our script: “read_tps_write_species_list_classifiers”. We then performed the nonphylogenetic geometric morphometric analyzes on each species using our custom script: “get_geomorphometric_sex_dimorphism_analyses”. Briefly, this algorithm reads the coordinate 2D‐array while excluding juvenile specimens and splitting adults into male and female groups (sex‐groups). Next, the algorithm calculates the Procrustes coordinates of each sex‐group landmark data, and the mean shape of aligned specimens within the sex‐group. With the resulting output, the following analyzes are implemented for each sex‐group: Procrustes ANOVA (with and without shape‐size covariation), morphological disparity (with and without shape‐size covariation, using overall mean and group means), and plots landmark coordinates (e.g., aligned specimen coordinates, and mesh deformation). All output for these analyzes is written in text and pdf files for further interpretation. For comparisons of body size (i.e., SVL) between sexes, we used the Welch two sample *t*‐test with gender as a grouping variable as implemented in the function “t.test” from R‐stats (R‐Core‐Team, 2016).

The phylogeny‐adjusted comparative analyzes were based on custom R‐scripts derived from “geomorph” ver 3.0.2 (Adams et al., 2016). These analyzes required the species means per landmark for each sex, which was estimated using the “get_geomorph_species_means” custom script. The input phylogeny was the Corytophanidae chronogram estimated in the previous section. We performed all phylogenetic geometric morphometric analyzes using the custom script: “get_phylogenetic_geomorphometric_analyses_by_sex.” Briefly, this algorithm reads the tree and the aligned landmark coordinates for each sex and species, and then estimates the phylogenetic signal for shape data using “physignal” function of “geomorph” ver 3.0.2 (Adams et al., 2016). The strength of the signal is returned as a multivariate K‐statistic (Kmult) adapted from Blomberg's K (Adams, 2014; Blomberg, Garland, & Ives, 2003). The algorithm then estimates a series of calculations on the shape data for each sex and between sex‐groups including: (1) the comparison of evolutionary rates, (2) phylogenetic integration, (3) phylogenetic modularity, and (4) phylogenetic ANOVA. These measurements of phylogenetic integrations and modularity at the intraspecific level were estimated under a phylogenetic context using evolutionary covariance matrices and implement in “geomorph” ver 3.0.2 (Adams, 2016; Adams & Felice, 2014). For the corytophanids, we tested for disparities in the rates of shape evolution between the lineages of the sexually dimorphic *Basiliscus* versus *Corytophanes *+ *Laemanctus* (both considered monomorphic). Likewise, we tested for phylogenetic morphological integration and modularity between the crest (Fig. S1, 9‐12 landmarks) versus the rest of the facial landmarks. Finally, the algorithm plots a phylogenetic tree and the Procrustes‐aligned specimens by each sex‐group in tangent space. The output of all of these analyzes is written in text and pdf files for further interpretation.

## 3 RESULTS

### 3.1 Phylogeny and chronogram of the Corytophanidae

**Figure 1:**
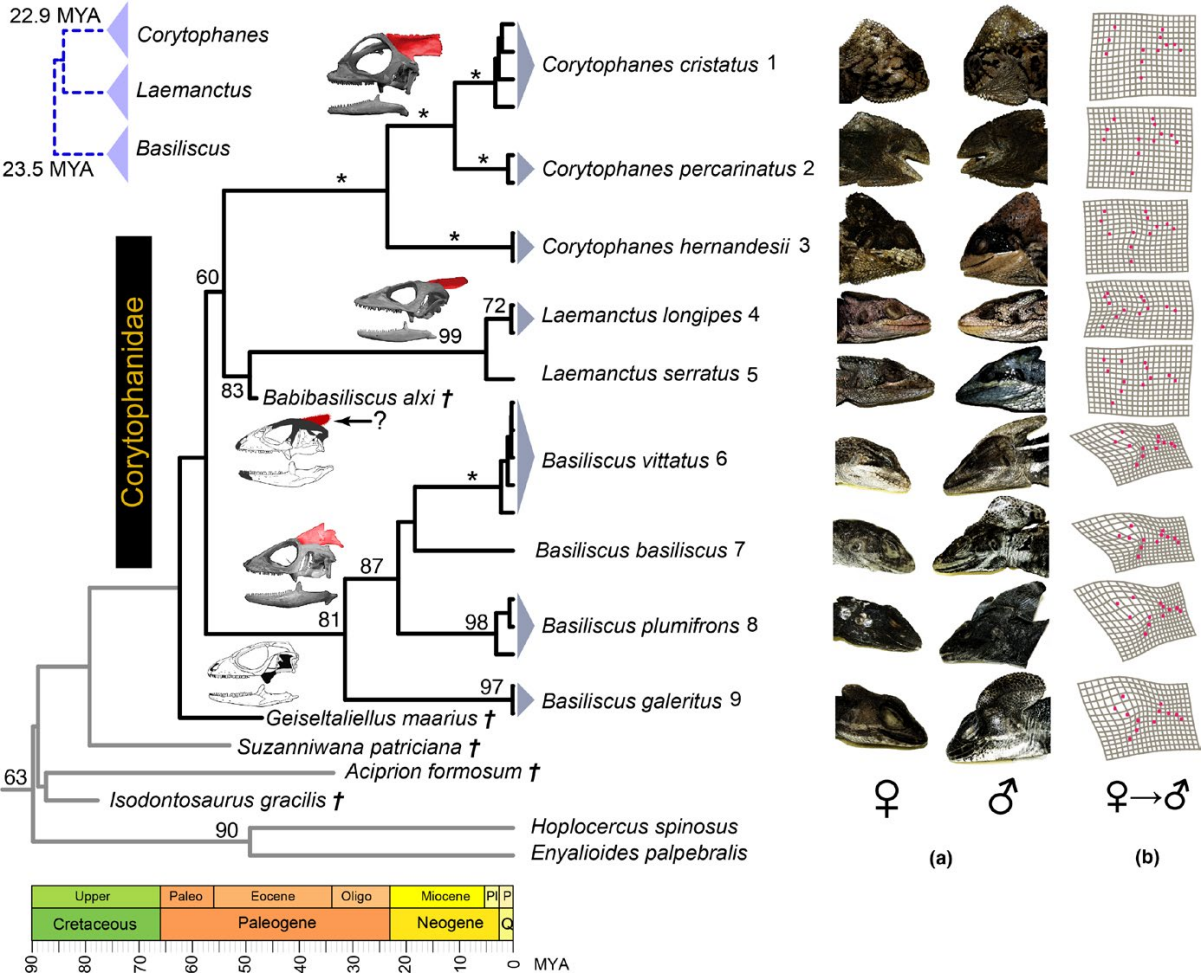
Time‐calibrated phylogeny of the casque‐headed lizards (Corytophanidae), and sexual dimorphism in head structure among extant species. (a) The sexual dimorphism in the head anatomy is evidenced by the crest structure, which is present in most members (=*basiliscus*,* plumifrons*, and *vittatus*) of *Basiliscus*. (b) Mesh deformation plots are the result of forcing female head landmarks into those of the males for each species; members of *Corytophanes* and *Laemanctus* show almost no deformation, while this is well developed in *Basiliscus*. The inclusion of the corytophanid fossils *Babibasiliscus alxi* and *Geiseltaliellus maarius* in the estimation of the chronogram has almost doubled the inferred ages of the crown and genus‐level divergences in Corytophanidae (previous estimates are indicated in the top‐left blue phylogeny). Nodal support (i.e., ML bootstrap support ≥ 60) is provided by values above lines and high support (i.e., ML support = 100) are indicated by an (*). The skulls are examples of each genus modified from (Conrad, 2015); the red overlay corresponds to the parietal crest bone and a question mark (?) indicates that the evidence of a crest is inconclusive. We propose two alternative evolutionary trajectories of this structure: ornamental (*Basilicus*) and biomechanical (*Corytophanes*); while this bone in *Laemanctus* shows an intermediate state

Our inferred phylogeny of the casque‐headed lizards (Figure 1) does not differ topologically from previous hypotheses (Blankers, Townsend, Pepe, Reeder, & Wiens, 2013; Lang, 1989; Vieira et al., 2005), with one exception; we recovered a (*Basiliscus basiliscus *+ *B. vittatus*) clade rather than a (*B. basiliscus *+ *B. plumiforms*) group resolved previously (Vieira et al., 2005). However, neither alternative topology is well supported (ML bootstrap < 70), and more molecular data are necessary. With respect to the placement of fossil taxa, *Babibasiliscus alxi* is supported as the sister taxon to the extant *Laemanctus* as previously reported (Conrad, 2015), but the corytophanid *Geiseltaliellus maarius* fossil is placed outside of, but sister to the Corytophanidae ingroup.

### 3.2 Geometric morphometrics and sexual dimorphism in Corytophanidae

At the intraspecific level, we compared the differences between shape of the cranial features between sexes. For instance, we compared the SVL between males and females of each species and only *Basiliscus* species (with the exception of *B. galeritus*) were sexually dimorphic (Table 1). For head morphology, we found that only the genus *Basiliscus* shows significant sexual dimorphism. In contrast, if allometry is accounted for, size contributes more than sex in head shape for all genera with the exception of *Laemanctus*. Specimens of this genus are rarer in collections, and only a small number of adult males (*n* = 2) were analyzed, which may not provide enough variation to estimate size contribution to sexual dimorphism in head shape. For *Basiliscus* species, only in *B. plumifroms* and *B. vittatus* did sex significantly influence differences in head shape. This result is best understood by looking at the sex disparity ratio in the last column of Table 1; this metric shows that among all species of *Basiliscus*, only *B*. *galeritus* has a low value. This result is because females of that species have relatively larger crests that in absolute size are only slightly smaller than those found on males.

At the family level, we use the interspecific phenotypic integration concept (see Section 1) to address how sexual dimorphism might differ across the casque‐headed lizard family. First, we found evidence of strong phylogenetic signal and significant differences in the rates of divergence and integration between crest structure and the rest of the face within the family Corytophanidae (Table 2). For instance, the *K*‐statistic showed that head shape had strong phylogenetic signal in both males and females across the family. In contrast, only males showed phylogenetic signal for head size. These results suggest that the size and shape of heads of males likely reflect the phylogenetic history of Corytophanidae. For females, head size tends to be relatively uniform across the family (i.e., it does not have significant phylogenetic signal), but head shape traces the phylogeny of corytophanids.

**Table 2:** Interspecific comparisons on head morphology by accounting for phylogenetic signal

Sexes	Phylogenetic signal (K)		Divergence rates		Integration: crest vs. not (r‐PLS)	Modularity: crest vs. not (CR)	D‐PGLS (*F*‐value)
	Shape	Size	Species	Landmarks			
♂	0.667**	0.884*	2.652^ns^	3.720**	.870^ns^	1.060^ns^	1.826^ns^
♀	0.835**	0.666^ns^	3.127^ns^	2.839*	.912*	1.227^ns^	1.189^ns^

Significance is indicated by: **p‐value < .01, *p‐value < .05, ^ns^ *p*‐value > .05.

“r‐PLS” refers to the mean of pairwise PLS correlations (*r*) between trait partitions, a measurement of phylogenetic morphological integration under Brownian motion model (Adams & Felice, 2014).

“CR” or Covariance Ratio refers to modularity signal between two trait modules of Procrustes‐aligned landmark coordinates in a phylogenetic context (Adams, 2016).

“D‐PGLS” refers to the results of Phylogenetic Procrustes ANOVA (Adams et al., 2016).

**Figure 2:**
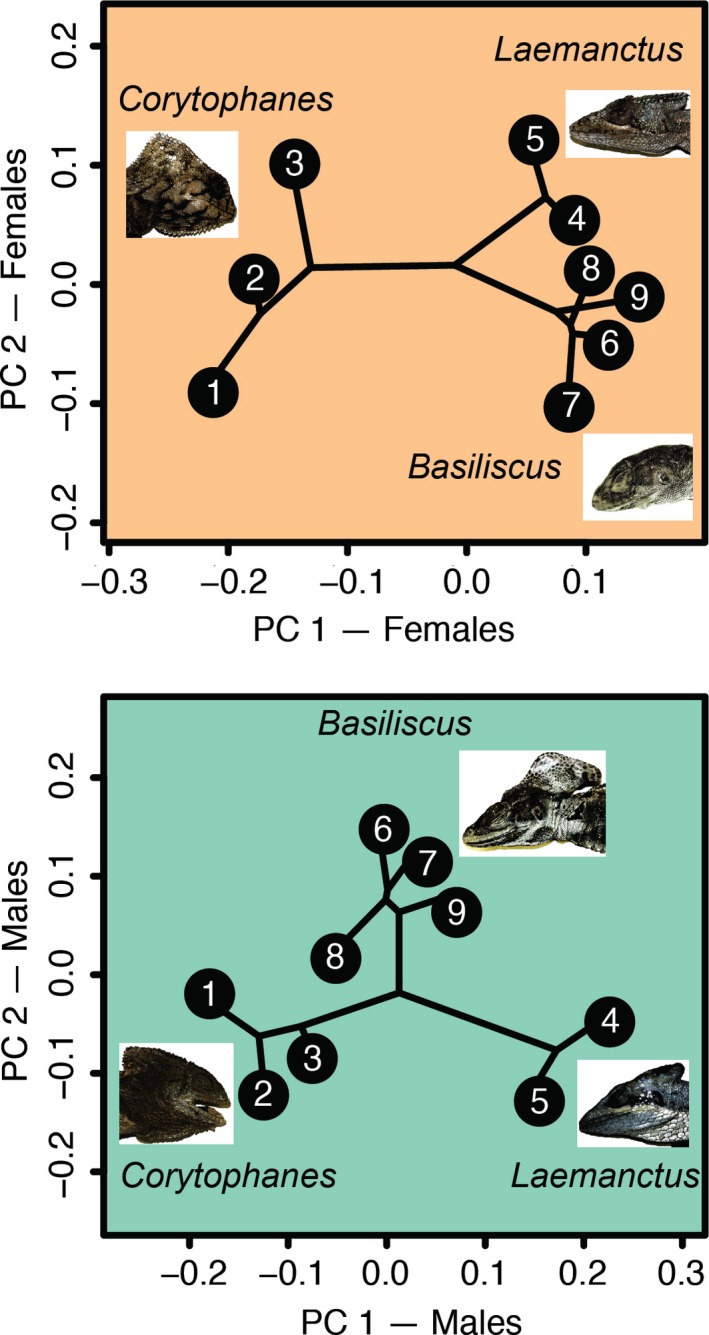
Principal dimensions of tangent space for male and female Procrustes‐aligned specimens. The phylogenetic tree is superimposed to reveal how head shape of Corytophanid lizards has evolved. On the female plot, *Laemanctus* and *Basiliscus* are more similar in shape than either is to *Corytophanes*. On the male plot, *Basiliscus* and *Corytophanes* are more similar in shape than either is to *Laemanctus*

Our inferences about sexual dimorphism were further evidenced in the principal component plots (Figure 2). In the females’ plot, *Basiliscus* and *Laemanctus* taxa are closer to each other in multivariate space than either is to female *Corytophanes* (which have crests). In the males’ plot, *Basiliscus* and *Corytophanes* taxa have developed crests and are closer to each other than either was to male *Laemanctus* (which do not have crests). Therefore, the three genera do not cluster in the same manner based on head morphology or on sex, revealing two types of sexual dimorphism. However, the extent to which the PCs summarize the differences in males versus females required the exploration of subsets of cranial landmarks.

Further analyzes showed that when comparing all 12 cranial landmarks between species there is no significant difference in head morphology, but when comparing the crest landmarks, the genus *Basiliscus* is significantly different from *Corytophanes* and *Laemanctus*. In contrast, a similar comparison showed no significant differences between these two latter genera. Therefore, when comparing the evolutionary rate of head morphology between *Basiliscus* and the other genera, *Corytophanes* and *Laemanctus* portray a higher rate of change in total head shape than does *Basiliscus*. In contrast, only the crest area in *Basiliscus* males exhibits a higher rate of change than males in the other two genera. Interestingly, the results of divergence rates for species and landmarks are consistent for both males and females. Consequently, our results for the comparison of phylogenetic integration between landmarks show that the head structure is integrated in females but not in males (Table 2). However, we found no evidence of modularity between crest landmarks and the rest of the head structure in either males or females.

## 4 DISCUSSION

We used geometric morphometric analyzes in combination with a time‐adjusted phylogeny of the Corytophanidae to show that significant sexual dimorphism in head structure is present in *Basiliscus*. In contrast, *Corytophanes* and *Laemanctus* have evolved a sturdier head structure in both sexes (i.e., monomorphic) during the same time period. Both evolutionary trajectories of the cranial structure data back to Eocene‐Oligocene at ~40 MYA and suggest that the casque‐headed lizards are a much older radiation than previously thought. Earlier studies based only on molecular markers placed the crown of Corytophanidae at 23.5 MYA in the Oligocene‐Miocene boundary. These node age discrepancies are common when chronograms are re‐estimated with the inclusion of unambiguous fossil ingroups.

Our estimated chronogram contrasts sharply with previous estimates for Corytophanidae mainly as a result of the temporal placement of the fossil species. Our inferred age of the crown of this family considering only extant taxa (and consistent with the divergence between *Basiliscus* and *Corytophanes+Laemanctus*) is 61.70 ± 5.12 MYA, which is 2.63 times the age proposed (i.e., 15.3–33.0 MYA; $\overset{¯}{X}=23.5\;\text{MYA}$) for this node from all previous studies (Blankers et al., 2013; Prates, Rodrigues, Melo‐Sampaio, & Carnaval, 2015; Townsend et al., 2011; Zheng & Wiens, 2016). This discrepancy is driven mainly by the placement of the *Babibasiliscus alxi* fossil which is dated at ~48 MYA (Conrad, 2015) and nested within Corytophanidae (Figure 1). Similarly, the placement of a putative corytophanid fossil lizard, *Geiseltaliellus maarius* outside of the corytophanid ingroup also provides evidence for a larger and more diverse, but now extinct, radiation of the Corytophanidae, and places the age of this group at least 66.82 ± 5.37 MYA (Fig. S2). Consequently, the divergence between *Corytophanes* and *Laemanctus* was inferred at 57.25 ± 3.87 MYA, which is 2.50 times the age previously proposed (i.e., 21.81–23.97 MYA; $\overset{¯}{X}=22.89\;\text{MYA}$) for this node (Blankers et al., 2013; Zheng & Wiens, 2016).

Following the best practices for justifying fossil calibrations (Parham et al., 2012), we consider that our older estimates of diversification in the Corytophanidae are better estimates of the chronology of this clade for the following reasons: (1) the corytophanid fossils included in the analyzes have clear provenance and expert identification; *B. alxi* UWBM 89090 (Conrad, 2015) and *G. maarius* HLND‐Me 10207 (Smith, 2009); (2) we included characters of both fossils in the 803‐character morphological matrix used in their phylogenetic estimation; (3) our tree topology that included these fossils is in agreement with the known molecular phylogeny of Corytophanidae; and (4) both fossils have a clear locality and stratigraphic level descriptions. For instance, we consider that the age of *B. alxi* is reliable as it comes from the well‐studied collection site “Lucky Lizard Locality,” Wyoming, USA, specifically from the Blacks Fork Member of Bridger B, Green River Basin dated in the late Eocene at ~48 MYA, as indicated in the original description (Conrad, 2015). For *G. maarius*, this fossil comes from the middle Messel Formation, dated at the middle Eocene (MP 11), within the Messel fossil Lagerstätte located near Frankfurt am Main (Germany), which is a UNESCO World Heritage site with exceptionally well‐preserved specimens (Smith, 2009). Overall, our estimated phylogeny provides new insights into a much older history of diversification associated with sexual dimorphism in light of our current understanding of the corytophanid fossil record.

Sexual dimorphism is common in lizards, and casque‐headed lizards (Corytophanidae) are no exception. In this clade, only males have strongly developed crests and large body sizes. However, a comparison among members of *Basiliscus* showed that only *B*. *plumifrons* and *B*. *vittatus* were sexually dimorphic when allometry was accounted for. The limited sexual disparity in *B. galeritus* is evidenced by the females having crests that resemble those of males, but the sexes differ in body size with females being smaller. In contrast, all species of *Corytophanes* are monomorphic for body size and both sexes have crests. The genus *Laemanctus* was also supported as monomorphic, but this may be an artifact of the small sample of males (*n* = 2 or 3), possibly insufficient to reveal sexual dimorphism. However, field observations and taxonomic accounts of this genus have not documented extreme sexual dimorphism between males and females (Lang, 1989), so further study of this issue is needed.

At the clade level, interspecific phenotypic integration is evidenced in the head shape features that together have strong phylogenetic signal in both sexes across Corytophanidae, while head size was only significant in males. This result suggests that size contributes to most head shape disparity between males and females, which may be an effect of allometric scaling on the morphological differences between sexes, and possibly even among species (Klingenberg, 2010). However, the phylogenetic perspective also suggests that male crests are evolving faster than the other head features. In contrast, female crests are evolving in concert with the rest of the head morphology, suggesting an underlying skull structure of the crest prone to sexual dimorphism. Our interpretation of all these results is that the crest morphology is evolving toward disparity in Corytophanidae, with two optima: ornamentation (sexually dimorphic) and feeding biomechanics (monomorphic).

Although the crest is evolving away from integration and faster than other head features, it does not necessarily evolve independently of other head characteristics. We provide further evidence of the heterogeneity in evolution of head dimensions by the plots of head shape by sex‐group (Figure 2). These results suggest that *Corytophanes* females have greater disparity in their head shape than the other genera, and a similar pattern is evident for *Basiliscus* males. This interpretation is supported by the observation that all *Corytophanes* females have a crest and that their head morphology is not much different from conspecific males. In contrast, *Basiliscus* males are very different from conspecific females as is evidenced by their larger and more distinctive crests. Therefore, we hypothesize that interspecific modularity is emerging in *Basilicus* between the crest and the rest of the head as this pattern of dimorphism of the male crest persists only within this genus.

Given the uniqueness of the crest over other head features, we propose two alternative evolutionary trajectories for crest function in the Corytophanidae: (1) the crest can be a signal or ornament associated with male status (Andersson, 1994), in any of the species in which sexual dimorphism is independent of allometry; and/or (2) the crest is an integrated structural component of the head related to feeding biomechanics (Johnston, 2014; Verwaijen & van Damme, 2007). Some evidence in favor of the ornamentation function exists for *Basiliscus*. In this genus*,* males are territorial and display active aggression toward smaller individuals, and reproduction is skewed in favor of larger males (Vandevender, 1978). The courtship in *Basiliscus* usually involves rapid vertical head motions (i.e., “head‐bobbing”) in which the crest becomes a prominent feature (Echelle & Echelle, 1972). Female mate choice and male aggressive interactions might then be hypothesized to drive the evolution and further development of the male crest, as in other examples of sexual ornamentation in lizards (Charles & Ord, 2012). Therefore, the evolution of a larger crest might “inflate” the body size image between competing males, and signal status to females during courtship in *Basiliscus*.

For the biomechanics hypothesis, morphological and functional evidence on the crest in *Corytophanes* suggest that this structure provides more area for the insertion of the feeding musculature (i.e., specifically the *M. adductor mandibulae externus medialis* and *M. pseudotemporalis* muscles; see (Schwenk, 1980)). If true, the bone structure of the crest would provides support for the musculature required for a greater bite force with larger gape angles, which would enable eating larger and chitinous prey items (Herrel, 2007; Miles, Losos, & Irschick, 2007). This inference is supported by diet accounts of *Corytophanes*, which largely specialize on adult coleopterans, orthopterans, and lepidopteran larvae (Andrews, 1979; Sasa & Monros, 2000). For *Laemanctus*, both of its extant species have the least developed crests in the Corytophanidae, but their structural resemblance to *Basiliscus* females favors an ornamentation function. However, more male specimens of *Laemanctus* are needed to further test our inference.

Given the phylogenetic position of *Corytophanes* and *Basiliscus*, we hypothesize that the ornamental and biomechanical functions of the crest are two alternative evolutionary trajectories of the parietal crest bones in the Corytophanidae. Our phylogeny suggests that the last ancestor of this lineage might have resembled the extinct *Geiseltaliellus maarius*. This fossil does not have a crest (see in (Conrad, 2015) fig. 5E), but it may have been a female, so this observation is of limited value. However, the absence of even a rudimentary crest suggests that at some point selection favored the development of extensions of the parietal bones, leading to more bone area for muscle insertion and a more powerful bite, as in *Corytophanes*. Consequently, we infer that the biomechanical function is a later evolutionary event, and might derive (i.e., as an exaptation) from the sexual ornamentation function of an ancestor with similar characteristics to an extant *Basiliscus*. In this genus, male competition and female choice select for larger body size, and the evolution of crests and fins in males. In the case of *Corytophanes*, diet specialization on large arthropods drives selection in favor of the evolution of a well‐developed crest in both sexes. Interestingly, the crest structure in *Laemanctus* is somewhat between these two alternatives, but it most closely resembles that of the sexually dimorphic *Basiliscus*. Further testing these hypotheses will require biomechanical, behavioral, and developmental data.

We provided definitions of phenotypic integration and modularity at the interspecific level. Phenotypic integration, the patterns or networks of highly correlated traits that persist across species and are evidenced by phylogeny‐adjusted correlations, constitute an operational definition of an integrated phenotype. Such networks of traits are inherited from ancestor to descendants and during this process new component traits can be integrated. Interspecific modularity is the expected outcome of selection during evolution of these phenotypic networks. In the case of sexual dimorphism, we found that male *Basiliscus* lizards present a highly dimorphic crest that can be associated with phenotypic integration. In contrast, both sexes of *Corytophanes* and *Laemanctus* present monomorphic head structure that is also phenotypically integrated and evolving toward a sturdier architecture that results in a more powerful bite.

## DATA ACCESSIBILITY

http://morphobank.org/permalink/?P2602,dryad.org. NCBI numbers: MF624292‐MF624309. Data available from the Dryad Digital Repository: https://doi.org/10.5061/dryad.r58r0

## CONFLICT OF INTEREST

None declared.

## AUTHORS’ CONTRIBUTIONS

GWT: Data collection, analysis and interpretation; drafting the manuscript. JCS: Conception or design of the work; data collection, analysis and interpretation; drafting and critical revision of the manuscript. BJP: Data collection. MM: Data collection; critical revision of the manuscript. CRVA: Data collection; critical revision of the manuscript. JWS: Conception or design of the work; analysis interpretation; drafting and critical revision of the manuscript.
