# Supplementary figures and images for: Sexual dimorphism, phenotypic integration, and the evolution of head structure in casque‐headed lizards
Source: Ecol Evol. 2017 Sep 25;7(21):8989–98. doi: 10.1002/ece3.3356 (PMC5689487; doi:10.1002/ece3.3356)

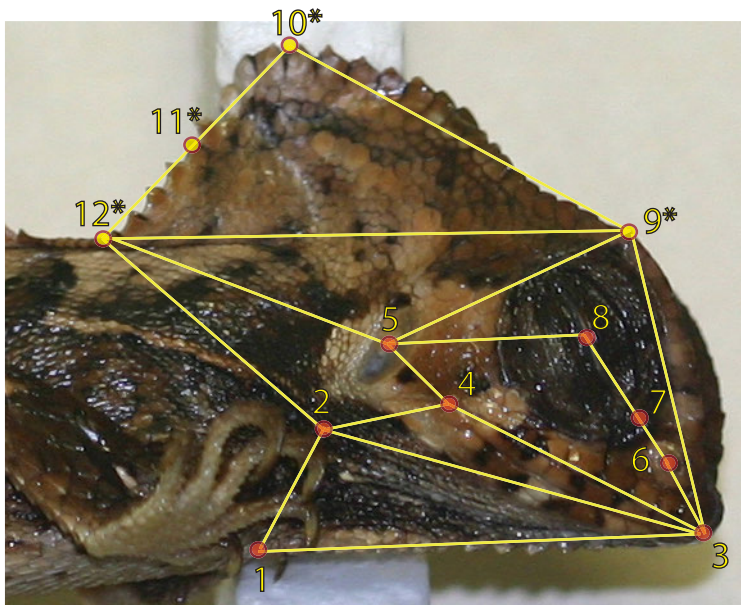

Figure S1

Supplement: Supplementary file 1 [file ECE3-7-8989-s001.pdf]

# Corytophanidae

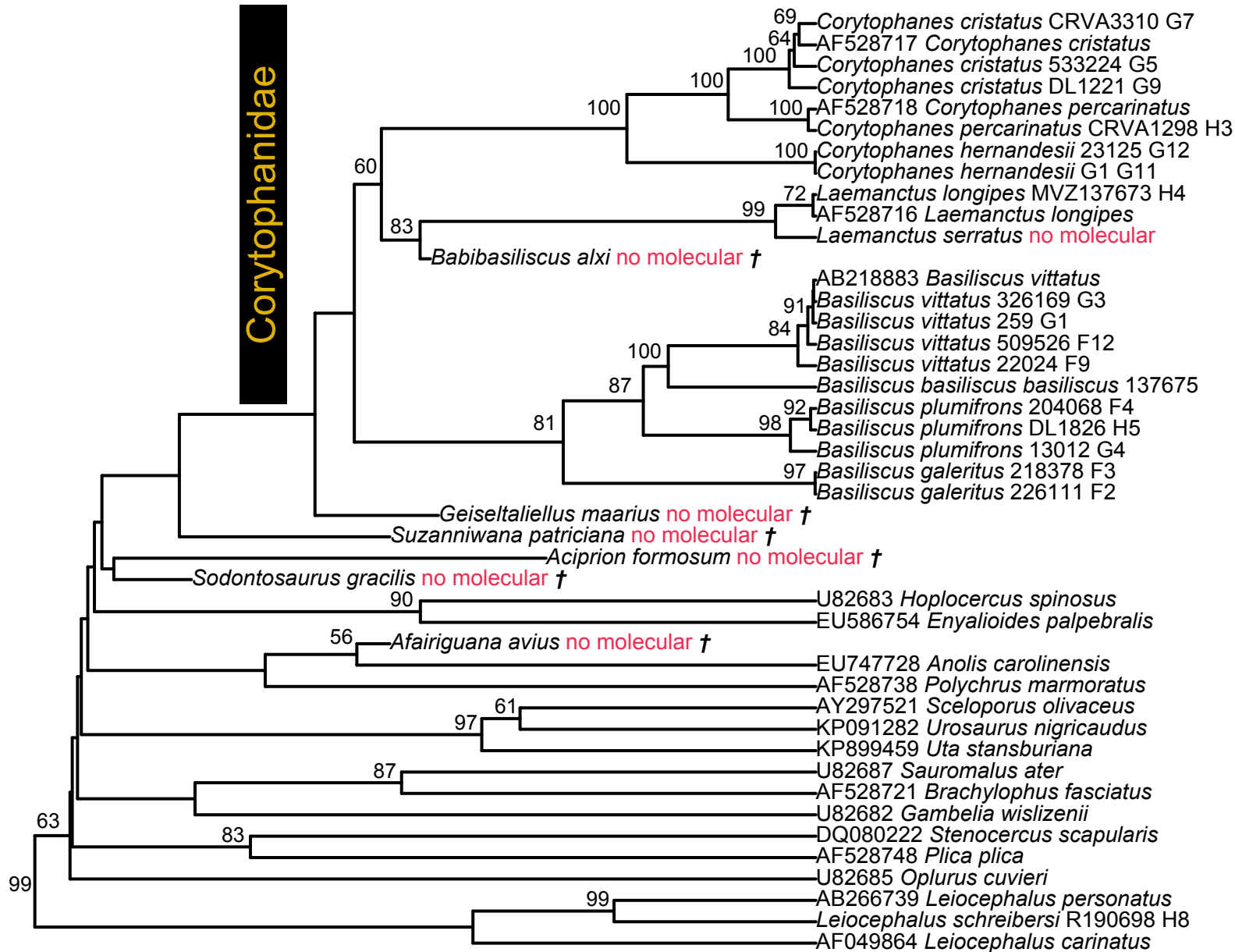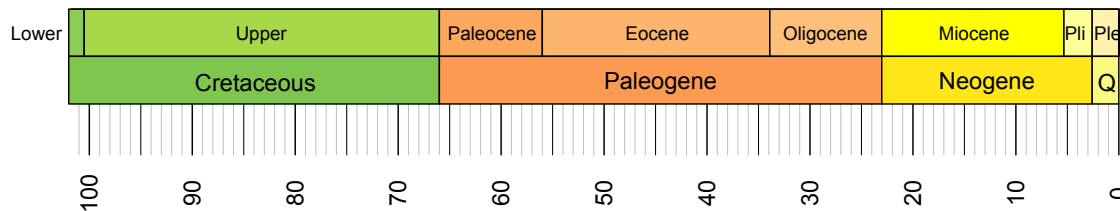

Supplement: Supplementary file 2 [file ECE3-7-8989-s011.pdf]
